# The NF‐κB‐signalling pathway in mare's endometrium infiltrated with the inflammatory cells

**DOI:** 10.1111/rda.14099

**Published:** 2022-02-25

**Authors:** Tomasz Jasiński, Łukasz Zdrojkowski, Ewa Kautz, Edyta Juszczuk‐Kubiak, Graça Ferreira‐Dias, Małgorzata Domino

**Affiliations:** ^1^ Department of Large Animal Diseases and Clinic Institute of Veterinary Medicine Warsaw University of Life Sciences (WULS – SGGW) Warsaw Poland; ^2^ Department of Molecular Biology Institute of Genetics and Animal Biotechnology of the Polish Academy of Sciences Jastrzębiec Poland; ^3^ Laboratory of Biotechnology and Molecular Engineering Department of Microbiology Prof. Wacław Dąbrowski Institute of Agricultural and Food Biotechnology—State Research Institute Warsaw Poland; ^4^ Departmento de Morfologia e Função CIISA ‐ Centro de Investigação Interdisciplinar em Sanidade Animal Faculdade de Medicina Veterinária Universidade de Lisboa Lisbon Portugal

**Keywords:** endometritis, HAS, IL‐6, mare, MCP‐1, NF‐κB

## Abstract

Endometritis is an important issue decreasing mares' fertility. In the case of endometritis, both inflammatory cells infiltration and proinflammatory molecules production are regulated by various cellular and gene regulatory mechanisms, including the nuclear factor‐κB (NF‐κB)‐dependent pathway. NF‐κB‐signalling pathway has been recently studied in the equine endometrium in the context of endometrosis. Thus, this study aimed to determine gene transcription of NF‐κB subunits (*RelA; NF‐κB1; NF‐κB2*), proinflammatory molecules (*MCP‐1; IL‐6*) and hyaluronan synthases (*HAS 1; HAS 2; HAS 3*) in endometritis and compare them with the intensity and type of inflammatory cell infiltration. Endometrial samples, collected post‐mortem from cyclic mares in oestrus or dioestrus, were classified histologically and examined using quantitative PCR. Transcription NF‐κB subunits genes did not differ with either inflammatory intensity or type of inflammatory cell infiltration. Transcription of *MCP‐1* and *IL‐6* genes increased with the severity of inflammation, with the involvement of *HAS 3* and *HAS 2* genes, as opposed to *HAS 1* genes. These proinflammatory molecules and hyaluronan synthases in the equine inflamed endometrium do not seem to be regulated by the NF‐κB pathway. Hence, separate signalling pathways for the development and progression of equine endometritis and endometrosis may be suggested.

## 1 INTRODUCTION

As mucous membranes are a part of the integumentary system, they play a crucial role in the protection of the underlying tissues from damage and infection. Thus, it is reasonable that, even in healthy tissue, inflammatory cells are present. While some infiltrate cells are sparsely distributed along with the tissue, some can be found in lymphatic follicles and around the endometrial glands, such as mononuclear inflammatory cell infiltrates interpreted as a regular component of the mucosa‐associated lymphoid tissue in submucous membranes (Klose & Schoon, 2016). This is the first barrier of an organism, while these cells are the first guardians against threats to homeostasis (Christoffersen & Troedsson, 2017). Mares’ endometrium may be facing bacterial influx during oestrus, after insemination/mating, delivery or even during diagnostic procedures, so the constant presence of limited infiltrate is legitimate. Apart from an immune role, these cells not also have a regulatory function, influencing fibroblasts, but also epithelium (Skarzynski et al., 2020).

Physiologically, neutrophils (polymorphonuclear cells, PMN) may be present in the lumen of a uterus only in oestrus, when the cervix is open and bacterial influx is possible. At that time, these cells may be found in the stratum compactum (SC) and in the lumen of endometrial glands (Overbeck et al., 2011). In addition, other types of cells, such as lymphocytes, macrophages and eosinophils, are in the endometrium in restricted amounts throughout the oestrous cycle. Most numerous inflammatory cells are lymphocytes, commonly seen not only in extracellular matrix (ECM) but also between epithelial cells (Watson & Thomson, 1996; Schöniger & Schoon, 2020). Macrophages and eosinophils are not so common in the endometrium. However, still, their presence in healthy endometrium is possible. There are always more cells in the SC of the endometrium, just below the luminal epithelium than in the stratum spongiosum (SP) (Watson & Thomson, 1996; Oddsdóttir et al., 2008; Overbeck et al., 2011; Schöniger & Schoon, 2020).

When the amount of the inflammatory cells infiltrating the uterine mucous membrane increases over the physiological level, endometritis develops (Morris et al., 2020). Endometritis is an important, often diagnosed issue decreasing mares' fertility. It can be divided into the acute post‐breeding form, characterized by a substantial increase in neutrophil presence in the endometrium, while in chronic inflammation, the number of lymphocytes considerably rises. While macrophages are presumed to regulate inflammation severity, eosinophils are present primarily in chronic endometritis. Regulatory mechanisms in inflammation are driven by cytokines, used for communication between cells in the endometrial microenvironment, as well as during inflammation activation. They may be produced by inflammatory cells, fibroblasts and epithelial cells. The most important cytokines in case of endometritis seem to be interleukins 1β, 6 and 10 (IL‐1β, IL‐6 and IL‐10), besides tumour necrosis factor α (TNF‐α). They are mostly proinflammatory, causing an increased influx of specific types of cells into the lumen of the uterus and endometrium itself (Schöniger & Schoon, 2020; Skarzynski et al., 2020). IL‐6 was found to play a predominant role in immune response in mares resistant to persistent breeding‐induced endometritis (PBIE), determining proper inflammatory reaction. It plays a role in the transition from acute to chronic inflammation, thus playing an important role as a modulator of inflammatory infiltration (Woodward et al., 2013). The release of IL‐6 is among others promoted by monocyte chemoattractant protein 1 (MCP‐1). Importantly, MCP‐1 itself is responsible for tissue migration and infiltration of immune cells in developing inflammation. Therefore, its expression is related to the intensity of inflammatory response in endometritis (Xu et al., 2015).

Both inflammatory cell infiltration and cytokine production are modulated by various cellular and gene regulatory mechanisms (Skarzynski et al., 2020). One of such mechanisms is the nuclear factor‐κB (NF‐κB)‐dependent pathway, which has been studied in the equine endometrium in the context of fibrosis or inflammation. NF‐κB transmission is involved in the synthesis of many proteins important in fibrotic, inflammatory and defence processes, regulating the expression of many different genes (May & Ghosh, 1998; Lind et al., 2001; Tripathi & Aggarwal, 2006; Domino et al., 2020). The NF‐κB family of proteins consists of five transcription factors, which can be divided into two groups in terms of their differences in structure, function and mechanism of formation. The first includes proteins: RelA (p65), RelB and cRel, while the second includes NF‐κB1 (p50/p105) and NF‐κB2 (p52/p100). The RelA, RelB and cRel proteins are characterized by the presence of transcription activation domain sequences, thanks to which they can activate the transcription of DNA molecules. The NF‐κB1 and NF‐κB2 proteins are synthesized as precursor proteins. As a result of proteolysis, active forms, p50 and p52, are formed. These proteins do not have domains necessary to activate transcription, however, when they form dimers with the RelA proteins, RelB and cRel are active forms (May & Ghosh, 1998). These active forms function as regulatory transcription proteins. Depending on the activating stimuli of the activation mechanism, the ways of activating NF‐κB proteins were distinguished as canonical and non‐canonical (Umezawa, 2011). The classic activation pathway depends mainly on the κB inhibitor kinase complex (IKK). IKKβ kinase phosphorylates nuclear factor of kappa light polypeptide gene enhancer in B‐cells inhibitor α (IκBα), which is ubiquitinated and degraded, thanks to which free p50/RelA and p50/c‐Rel dimers are transported from the cytoplasm to the cell nucleus. An alternative activation route depends on the IKKα subunit, a part of the IKK complex, which phosphorylates the p100 protein, which is part of the p100/RelB dimer, which is still transcriptionally inactive. The phosphorylated p100 protein is ubiquitinated, the end of the protein chain is degraded and the p52 protein is being formed. The active p52/RelB dimer penetrates the cytoplasm and influences the transcription of specific genes (May & Ghosh, 1998; Lind et al., 2001; Domino et al., 2020).

The NF‐κB pathway is thought to be crucial in the development of immune response, both by stimulating and inhibiting gene expressions in various cell types. Yet, any dysregulation of this system results in immune response disturbance. Interestingly, in the equine endometrium, both types of NF‐κB activation are suspected to downregulate IL‐6, having multiple functions in inflammation regulation (Tripathi & Aggarwal, 2006; Domino et al., 2020). Cytokines are not the only proinflammatory products of NF‐κB activation. Hyaluronan synthases (HAS 1, 2 and 3) are suspected to increase cell migration and adhesion, which also affect immune cell infiltration. Their properties influence the early stage of inflammation and modulate it (Necas et al., 2008). Additionally, HASs, IL‐6 and MCP‐1 seem to play an important role in the pathogenesis of endometrosis, another crucial reproductive problem in mares (Aresu et al., 2012). Endometritis and inflammation regulators are suspected to have a part in endometrosis pathogenesis, or even to be the main cause (Aresu et al., 2012; Rebordão, Carneiro, et al., 2014; Rebordão et al., 2018; Skarzynski et al., 2020).

Those recent findings raise the problem of NF‐κB‐signalling effect on leukocyte infiltration in the equine endometrium. Thus, this study aimed to compare gene transcription of NF‐κB subunits (*RelA; NF‐κB1; NF‐κB2*), proinflammatory molecules (*MCP‐1; IL‐6*) and hyaluronan synthases (*HAS 1; HAS 2; HAS 3*) depending on the inflammatory infiltration intensity and the type of inflammatory cell infiltration.

## 2 MATERIALS AND METHODS

### 2.1 Samples collection

Biological material for this study consisted of equine internal genitalia and blood. Samples were collected from 187 Polish warm‐blood mares (aged from 3 to 25 years) at a commercial abattoir in Poland. Samples were collected post‐mortem in reproductive season from April to September so that no Ethical Committee's permission was needed. According to decision of II Local Committee for Ethics in Animal Research WULS in Warsaw, this type of project does not fall under the legislation for the protection of animals used for scientific purposes (national decree‐law, Dz. U. 2015 poz. 266 and EU law, 2010‐63‐EU directive). Equine internal genitalia included ovaries and two endometrial samples from the uterine body. Samples were collected immediately after evisceration. Ovaries were placed into containers with cold saline (0.9% NaCl, Polfa S.A., Poland), transported at +4°C to the laboratory and sectioned to note the presence and diameter of follicles and/or corpus luteum. Endometrial samples were collected for histological examination and gene transcription analyses. Samples for histological examination were collected into container with the 10% formalin (Sigma‐Aldrich, Poland), fixed routinely and embedded in paraffin equivalent (Sigma‐Aldrich, Poland). Sample for gene transcription analyses was collected into RNase‐free Eppendorf tubes (Eppendorf AG, Germany), snap‐frozen in liquid nitrogen and stored at −80°C. Blood samples were collected into dry tubes (BD Vacutainer®, UK), transported to the laboratory at +4°C and centrifuged (2,000x g, 5 min). The serum free from any apparent haemolysis was aspirated and stored at −20°C for further hormone's analyses.

### 2.2 Histopathological examination

Endometrial samples in paraffin blocks were cut in 5 μm sections (rotation microtome Leica RM2255; Kawa‐Ska, Polska) and mounted on silanized glass slides. Standard histological staining procedure was performed including deparaffinization, rehydratation, stained in automated slide stainer (Multistainer Leica ST5020; Kawa‐Ska, Polska) with haematoxylin (3801520E, Leica, United States) and eosin (HT1103128; Sigma Aldrich, Poland) (HE staining) and mounted under Canadian balsam (Sigma‐Aldrich, Poland). Stained slides were examined under a light microscope (Olympus BX43, Poland) to assess the presence of inflammation and the appearance or severity of pathological degenerative changes. Each endometrial sample was triple categorized using two endometritis criteria (the intensity and the type of inflammatory cell infiltration) and the oestrous cycle criterion (the follicular phase, FLP, and the mid‐luteal phase, MLP). Endometrial samples were classified as presenting endometritis or not (control group) focusing on the SC. Three groups regarding the intensity of inflammatory cell infiltration, and independent from the four groups of different types of inflammatory cell infiltration, were created. The intensity of inflammatory cell infiltration was quantified as mild infiltration, moderate infiltration or severe infiltration, whereas the type of inflammatory cell infiltration was classified as macrophages infiltration, neutrophil infiltration, lymphocytes infiltration or mixed infiltration based on the type of cells recognized in the histologic sections. Neutrophil and macrophage mild infiltration was considered when 1–2 cells were depicted per 5 microscopic fields; moderate infiltration when 3–4 cells were present and severe infiltration showed >5 cells (Ricketts & Alonso, 1991; Summerfield & Watson, 1998). For lymphocyte infiltration, evenly scattered lymphoid cells were the grounds for classification as mild infiltration, while addition of aggregates was seen in moderate infiltration, and multiple aggregates, with more lymphoid cells than fibroblasts in SC, were recognized as severe infiltration (Watson & Thomson, 1996; Nervo et al., 2019). Mixed infiltration was considered when at least two types of cells with moderate level of infiltration were present in endometrium. When the microscopic hallmarks of endometrosis were observed (Schöniger & Schoon, 2020), the sample was excluded from the studied group. Among 187 collected samples, 67 samples were excluded based on the endometrosis recognition and 120 samples were classified as endometrosis free. Only endometrosis‐free samples were qualified for gene transcription evaluation. Among 120 qualified samples, 24 samples of healthy endometrial tissue were included in the control group (*n* = 24). Half of the samples were collected from mares in FLP (*n* = 12) and the other half in MLP (*n* = 12) of the oestrous cycle. The remaining 96 samples were assigned to each of the three groups of the intensity of inflammatory cell infiltration, as follows: (i) mild infiltration (*n* = 32), (ii) moderate infiltration (*n* = 32) or (iii) severe infiltration (*n* = 32). In addition, independently from each of these four groups, equine endometrial histological samples were categorized according to the type of inflammatory cell infiltration, as follows: (i) macrophages infiltration (*n* = 24), (ii) neutrophil infiltration (*n* = 24), (iii) lymphocytes infiltration (*n* = 24) or (iv) mixed infiltration (*n* = 24). In all groups, half of the endometrial samples were collected from mares in FLP and grouped depending on the infiltration intensity (*n* = 16 samples from each group); or on the infiltration type (*n* = 12 samples from each group). Likewise, the other half of the endometrial samples were from mares in MLP and were classified according to the infiltration intensity (*n* = 16 samples from each group); or on the infiltration type (*n* = 12 samples from each group). Part of the results on the transcription of selected genes in healthy endometrial tissue were previously documented (Domino et al., 2020).

### 2.3 Gene transcription evaluation

Frozen endometrial samples were mechanically disrupted in liquid nitrogen environment and then homogenized (50 mg of each sample; Lysing Matrix D tubes, MP Biomedicals, United States). The total RNA was extracted using the standard protocol (High Pure RNA Tissue kit; Roche, Switzerland). A DNase treatment was performed, and the RNA concentration was determined with absorbance ratios A260/280 and A260/230 (DS‐11 FX spectrophotometer; DeNovix, United States). All samples evaluated in this study had a high enough RNA concentration, beyond 100 ng. In each endometrial sample, transcription of the following eight genes was assessed by real‐time PCR (qPCR): *RelA, NF‐κB1, NF‐κB2, MCP‐1, IL‐6, HAS 1, HAS 2* and *HAS 3*. The qPCR amplification was performed using a TaqMan™ RNAto‐CT™ 1‐Step kit (No 4392938, ThermoFisher, United States) and a Quant‐Studio™ 6 Flex Real‐Time PCR System (Applied Biosystems, United States). The commercially available equine‐specific TaqMan Gene Expression Assays (No 4448892 and 4441114, ThermoFisher, United States) were used. The list of primers and 6‐carboxyfluorescein (6‐FAM) and 6‐carboxytetramethylrhodamine (TAMRA)‐labelled TaqMan probes was previously documented (Domino et al., 2020) and listed in Table 1. The qPCR reaction (10 ml volume per each reaction) included as follows: (i) 15 ng of total RNA, (ii) 5 ml of TaqMan® RTPCR Mix (2X), (iii) 0.25 ml of TaqMan® RT Enzyme Mix (40X), (iv) 0.5 ml of TaqMan probe and (v) both PCR primers (ThermoFisher, USA) for each gene of interest. The qPCR protocol included four steps as follows: (i) reverse transcription (15 min at 48°C), (ii) enzyme activation (10 min at 95°C), (iii) 40 cycles of denaturation (15 s at 95°C) and (iv) annealing/extension (1 min at 60°C). Each sample was run in triplicate. Raw data of genes transcription were normalized using the geometric mean of reference genes (GAPDH, HPRT1). The semi‐quantitation of the target gene expression was performed in a comparative CT method (ΔΔCT method), where the target gene expression in the samples of control group was considered as ΔCt control value.

**TABLE 1:** List of primers and TaqMan probes used for the qRT‐PCR analysis

Gene	TaqMan assay ID/primers/probe	Amplicon length (bp)
*RelA (p65)*	Bt03253757_m1	59
*NK‐κβ1 (p105/p50)*	Bt03243456_m1	107
*NK‐κβ2 (p100/p52)*	Bt03272792_g1	101
*MCP‐1 (CCL2)*	Ec03468496_m1	66
*IL‐6*	Ec03468680_m1	117
*HAS1*	F:GCCTATCAGGAACAACCTCC R: TAGCCCATGCTGAGCATC P: ACAACCAGAAGTTCCTGGGCACCCAC	124
*HAS2*	Ec03467769_m1	63
*HAS3*	Bt04298490_m1	73
*GAPDH*	Ec03210916_gH	118
*HPRT1*	Ec03470217_m1	84

### 2.4 Phases of oestrous cycle determination

In stored serum samples, the progesterone (P4) concentration was determined using a commercial radioimmunoassay (KIP 1458; DIAsource ImmunoAssays SA, Belgium; intra‐assay coefficient of variation <5.6%; inter‐assay coefficient of variation <8.8%). The standard protocol was used and the absorbance was measured by Multiscan Reader (Labsystem, Finland) Genesis and V 3.00 software. The P4 concentration and the ovaries macroscopic view were used to determinate the phases of the oestrous cycle, following da Costa et al. (2007) protocol. Mares were assigned into FLP group when P4 concentration was <1 ng/ml, and at least one follicle >35 mm in diameter in any of the ovaries, and no corpus luteum present was demonstrated. Mares were assigned in MLP group when P4 concentration was >1 ng/ml and on both ovaries, none of the follicles >35 mm in diameter and at least one corpus luteum was demonstrated. Among 120 endometrial samples, 60 samples were qualified as belonging to MLP mares (*n* = 60) or FLP mares (*n* = 60) groups. None of the samples was excluded due to the failure phase determination.

### 2.5 Statistical analysis

Univariate marginal distributions of normalized qPCR data were tested independently for each endometrial samples group and each target gene using a univariate Kolmogorov–Smirnov test. Because of the non‐Gaussian distribution of at least one data series in each data being compared, the comparisons within the intensity of inflammatory cell infiltration groups and the type of inflammatory cell infiltration groups were assessed by Kruskal–Wallis test, followed by Dunn’s multiple comparisons test. The comparisons between phases of the oestrous cycle were performed by unpaired *t* test with Welch’s correction for Gaussian data pairs or Mann–Whitney test for non‐Gaussian data pairs. Numerical data were reported on plots as mean +standard deviation (SD). All statistical analyses were performed using GraphPad Prism6 software (GraphPad Software Inc., USA), where the significance level was established as *p*<0.05.

## 3 RESULTS

**FIGURE 1:**
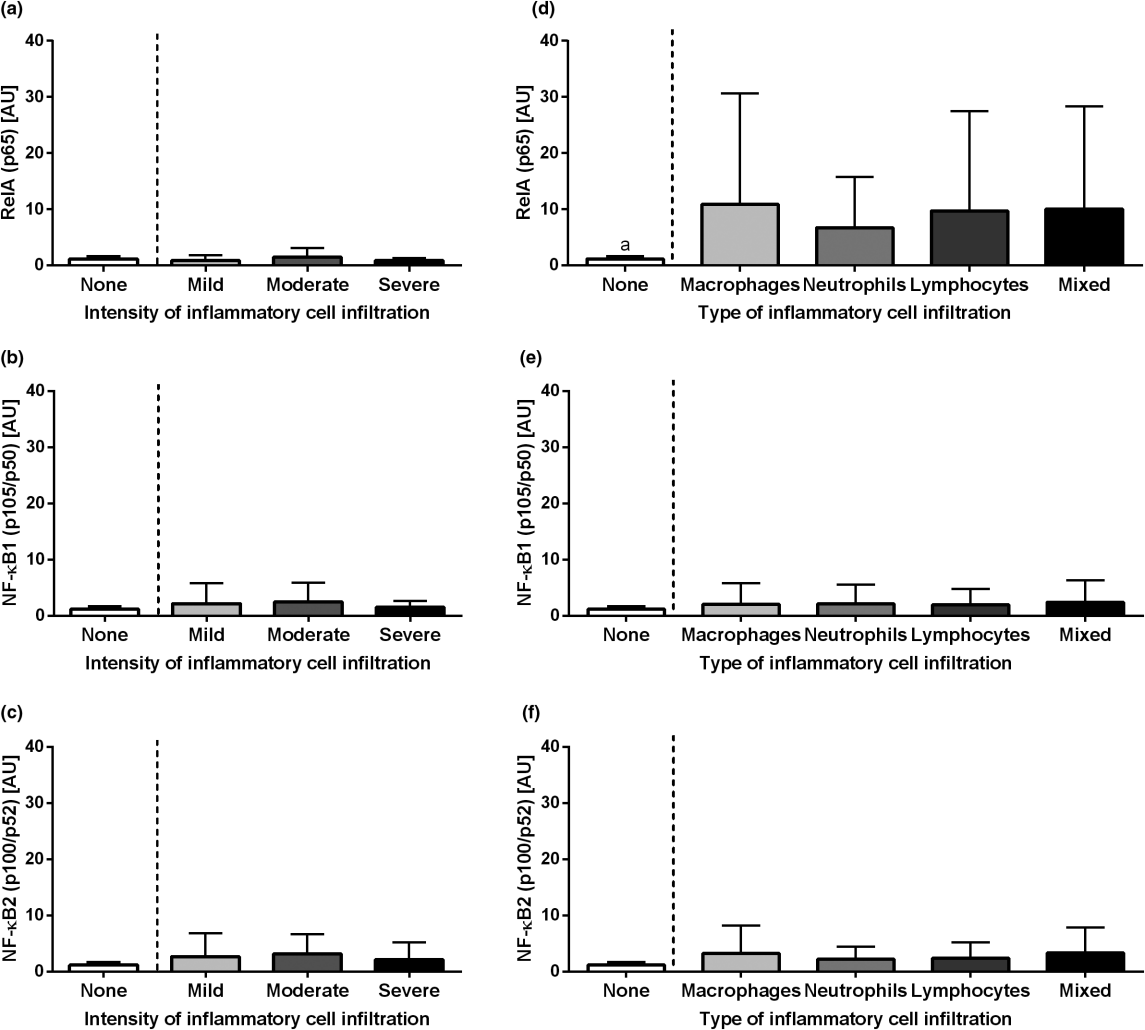
The transcription levels (mean + SD) of the nuclear factor‐κB subunit RelA (*RelA*; *p65*) (a, d); of nuclear factor‐κB subunit 1 (*NF‐κB1*; *p105/p50*) (b, e) and of nuclear factor‐κB subunit 2 (*NF‐κB2*; *p100/p52*) (c, f) in the mares' endometrium. The endometrial samples evaluated with respect to the intensity of inflammatory cell infiltrate (none, mild, moderate and severe) are shown in graphs a, b and c, and according to the type of inflammatory cell infiltration (macrophages, neutrophils, lymphocytes and mixed cells) or no cell infiltration detected (none) in d, e and f. No significant differences were found between groups (*p *> .05)

The transcription of the genes of NF‐κB subunits under study did not differ in equine endometrium depending on the intensity of inflammatory cell infiltration for both the canonical (*RelA*, *p *= .1, Figure 1a; *NF‐κB1*, *p *= .8, Figure 1b) and the non‐canonical (*NF‐κB2*, *p *= .8, Figure 1c) pathways. Similarly, the transcription of the genes *RelA* (*p *= .2, Figure 1d), *NF‐κB1* (*p *= .9, Figure 1e) and *NF‐κB2* (*p *= .9, Figure 1f) did not differ with the type of inflammatory cell infiltration.

**FIGURE 2:**
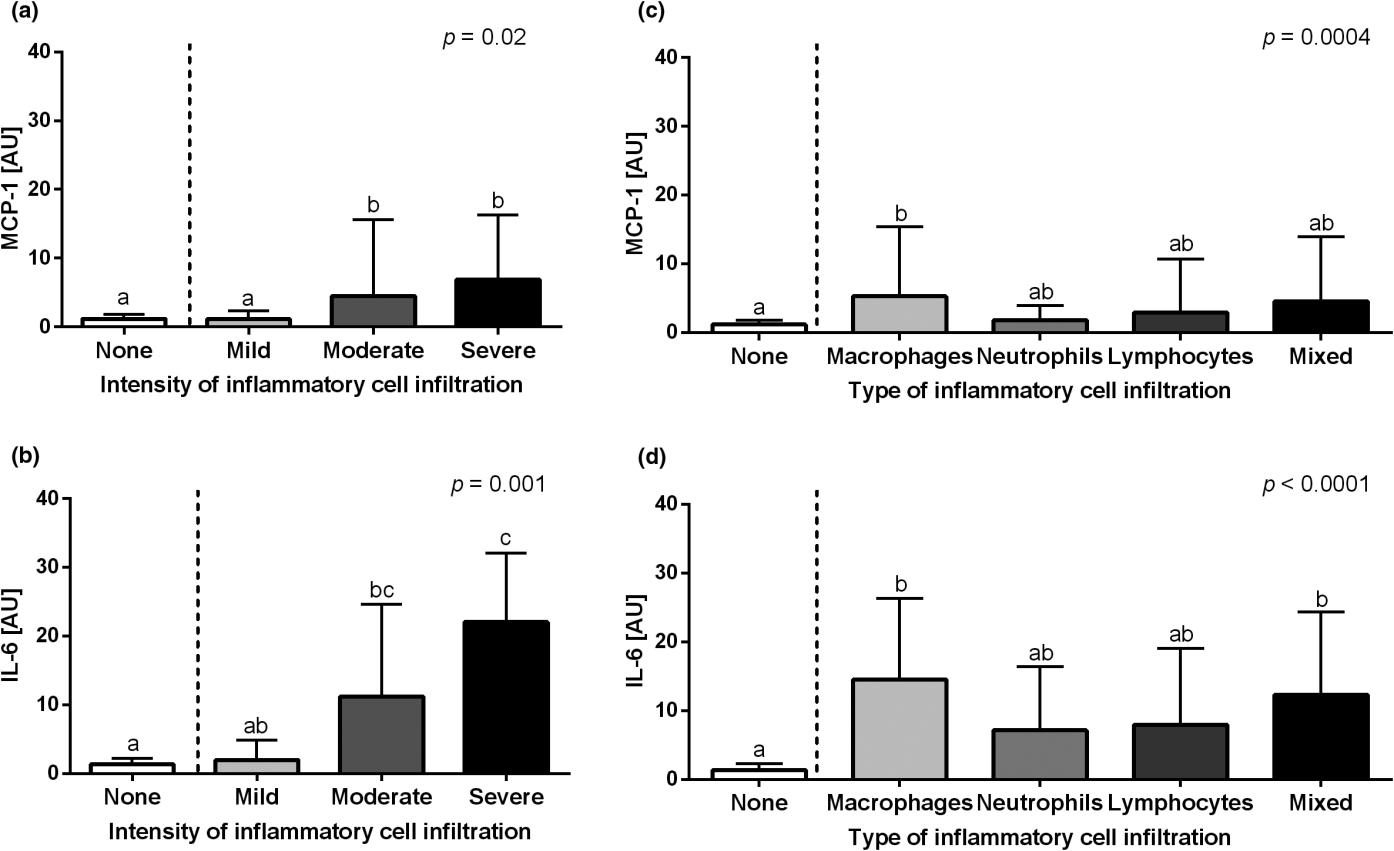
The transcription levels (mean + SD) of (a, c) the monocyte chemoattractant protein‐1 (*MCP‐1*) and (b, d) interleukin‐6 (*IL‐6*) in the mares' endometrium. The endometrial samples evaluated with respect to the intensity of inflammatory cell infiltrate (none, mild, moderate and severe) are shown in graphs A and B, and according to the type of inflammatory cell infiltration (macrophages, neutrophils, lymphocytes and mixed cells) or no cell infiltration detected (none) in C and D. Different letters indicate differences between groups for *p *< .05

The transcription of *MCP‐1* gene was higher in equine endometrium with the moderate and severe intensity of inflammatory cell infiltration than in the mild‐intensity infiltration group and the control group (*p *= .02, Figure 2a), whereas the transcription of *IL‐6* gene increased gradually with the intensity infiltration. Compared to the control group, *IL‐6* gene transcription was higher in the moderate‐intensity infiltration group and the highest in the severe‐intensity infiltration group with no differences between moderate and severe groups, mild and moderate groups as well as control and mild groups (*p *= .001, Figure 2b). Moreover, the transcription of both *MCP‐1* (*p *= .0004, Figure 2c) and *IL‐6* genes was higher in equine endometrium with macrophages infiltration than in the control groups (*p *< .0001, Figure 2d), with no differences with the neutrophil, lymphocytes and mixed types of inflammatory cell infiltration. Additionally, the transcription of *IL‐6* gene was also increased in the equine endometrium with mixed infiltration than in the control groups.

**FIGURE 3:**
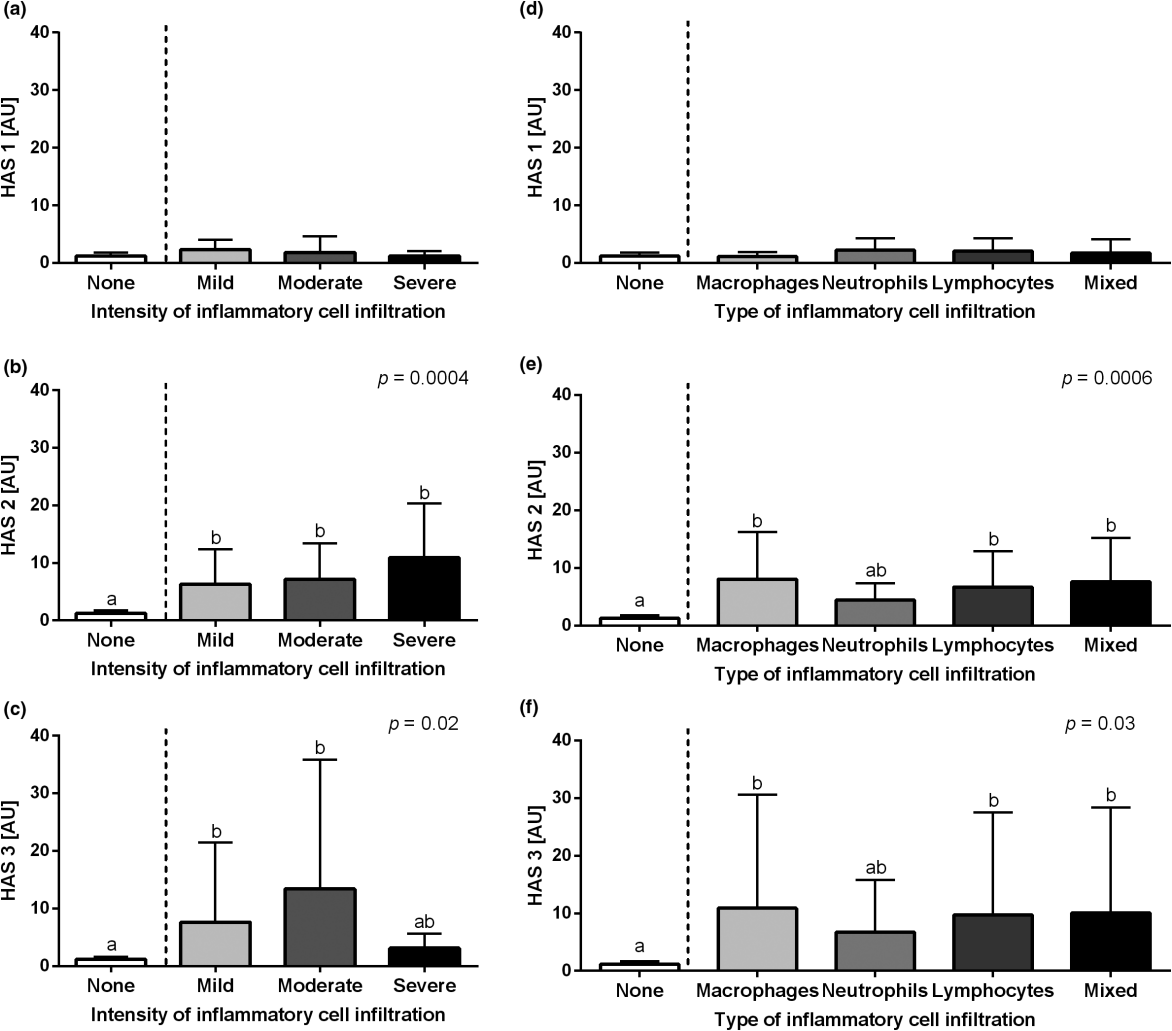
The transcription levels (mean + SD) of (a, d) the hyaluronan synthase 1 (*HAS 1*), (b, e) hyaluronan synthase 2 (*HAS 2*) and (c, f) hyaluronan synthase 3 (*HAS 3*) in the mares' endometrium. The endometrial samples evaluated with respect to the intensity of inflammatory cell infiltrate (none, mild, moderate and severe) are shown in graphs A, B and C, and according to the type of inflammatory cell infiltration (macrophages, neutrophils, lymphocytes and mixed cells) or no cell infiltration detected (none) in D, E and F. Different letters indicate differences between groups for *p *< .05

The transcription of the HASs genes under study differed depending on both type of HAS and the inflammatory status of endometrium. The transcription of *HAS 1* gene did not differ in equine endometrium depending on both the intensity (*p *= .1, Figure 3a) and the type (*p *= .8, Figure 3d) of inflammatory cell infiltration. On the other hand, the transcription of *HAS 2* gene was higher in equine‐infiltrated endometrium than in the control group regardless of the intensity infiltration and with no differences among mild, moderate and severe groups (*p *= .0004, Figure 3b). Similarly, the transcription of *HAS 3* gene was higher in equine endometrium with the mild‐ and moderate‐intensity infiltration than in the control group, but not in the severe infiltration group. No differences were noted for the *HAS 3* gene transcription between the control and severe groups, as well as mild, moderate and severe groups (*p*=0.02, Figure 3c). The transcription of both *HAS 2* (*p* = .0006, Figure 3e) and *HAS 3* (*p *= .03, Figure 3f) genes was higher in equine endometrium with macrophages, lymphocytes and mixed infiltration than in the control groups. Yet, no differences between the control and neutrophil infiltrated groups as well as between groups with different types of inflammatory cell infiltration were noted.

**FIGURE 4:**
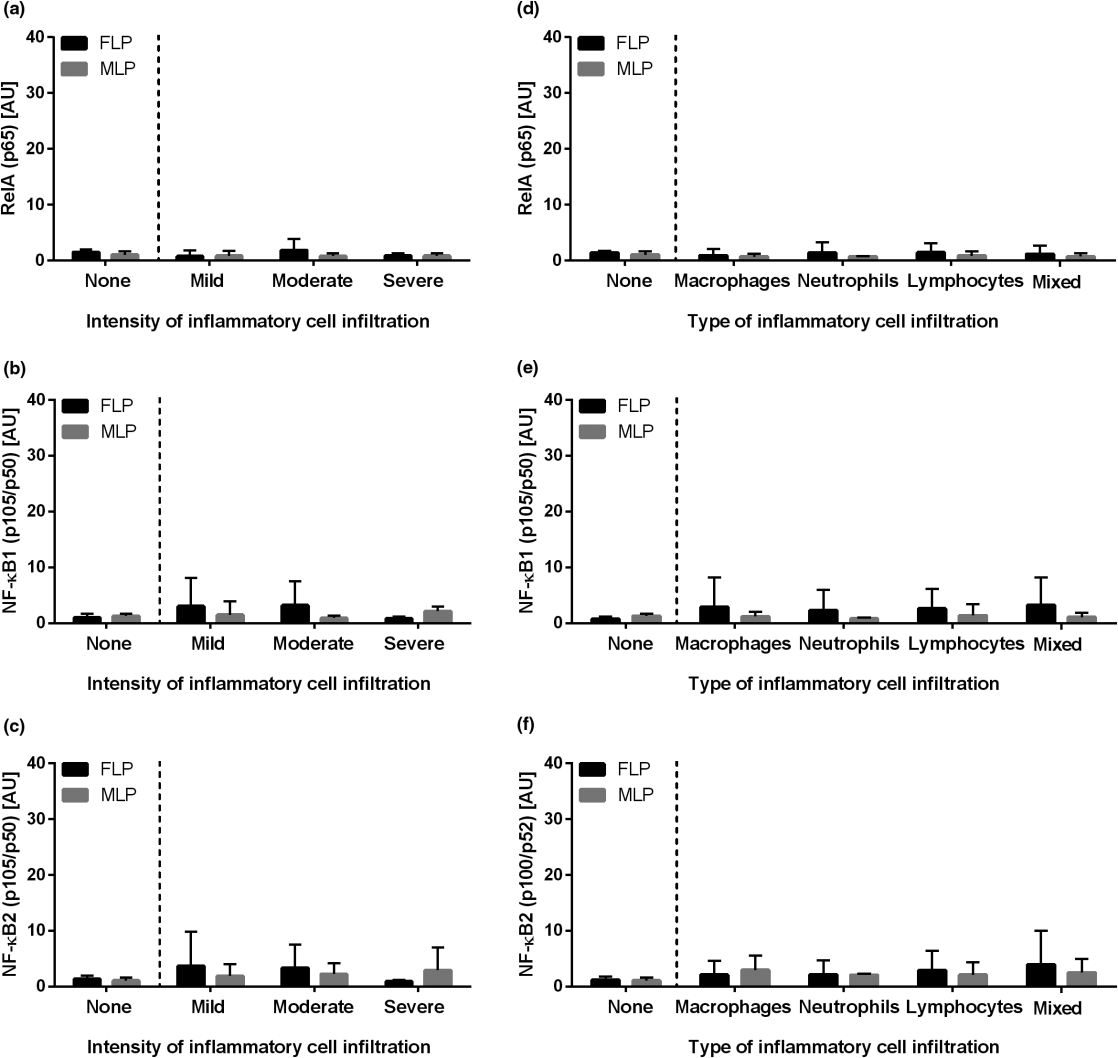
The transcription levels (mean + SD) of (a, d) the nuclear factor‐κB subunit RelA (*RelA*; *p65*), (b, e) nuclear factor‐κB subunit 1 (*NF‐κB1*; *p105/p50*) and (c, f) nuclear factor‐κB subunit 2 (*NF‐κB2*; *p100/p52*) in the mares' endometrium in follicular (FLP) or mid‐luteal (MLP) phases of the oestrous cycle. The endometrial samples evaluated with respect to the intensity of inflammatory cell infiltrate (none, mild, moderate and severe) are shown in graphs a, b and c, and according to the type of inflammatory cell infiltration (macrophages, neutrophils, lymphocytes and mixed cells) or no cell infiltration detected (none) in d, e and f. No differences were found between the phases of the oestrous cycle for any of the studied NF‐κB subunits (*p *> .05)

When the phases of the oestrous cycle were considered, as before the transcription of the genes of NF‐κB subunits did not differ in equine endometrium depending on the intensity of inflammatory cell infiltration for both the canonical (*RelA*, FLP *p *= .4, MLP *p *= .4, Figure 4a; *NF‐κB1*, FLP *p *= .5, MLP *p *= .06, Figure 4b) and the non‐canonical (*NF‐κB2*, FLP *p *= .9, MLP *p *= .7, Figure 4c) pathways. Likewise, the transcription of the genes of *RelA* (FLP *p *= .3, MLP *p *= .6, Figure 4d), *NF‐κB1* (FLP *p *= .9, MLP *p *= .5, Figure 4e) and *NF‐κB2* (FLP *p *= .996, MLP *p *= .6, Figure 4f) did not differ depending on the type of inflammatory cell infiltration. No differences (*p *> .05) were found between the phases of the oestrous cycle for any of the studied NF‐κB subunits.

**FIGURE 5:**
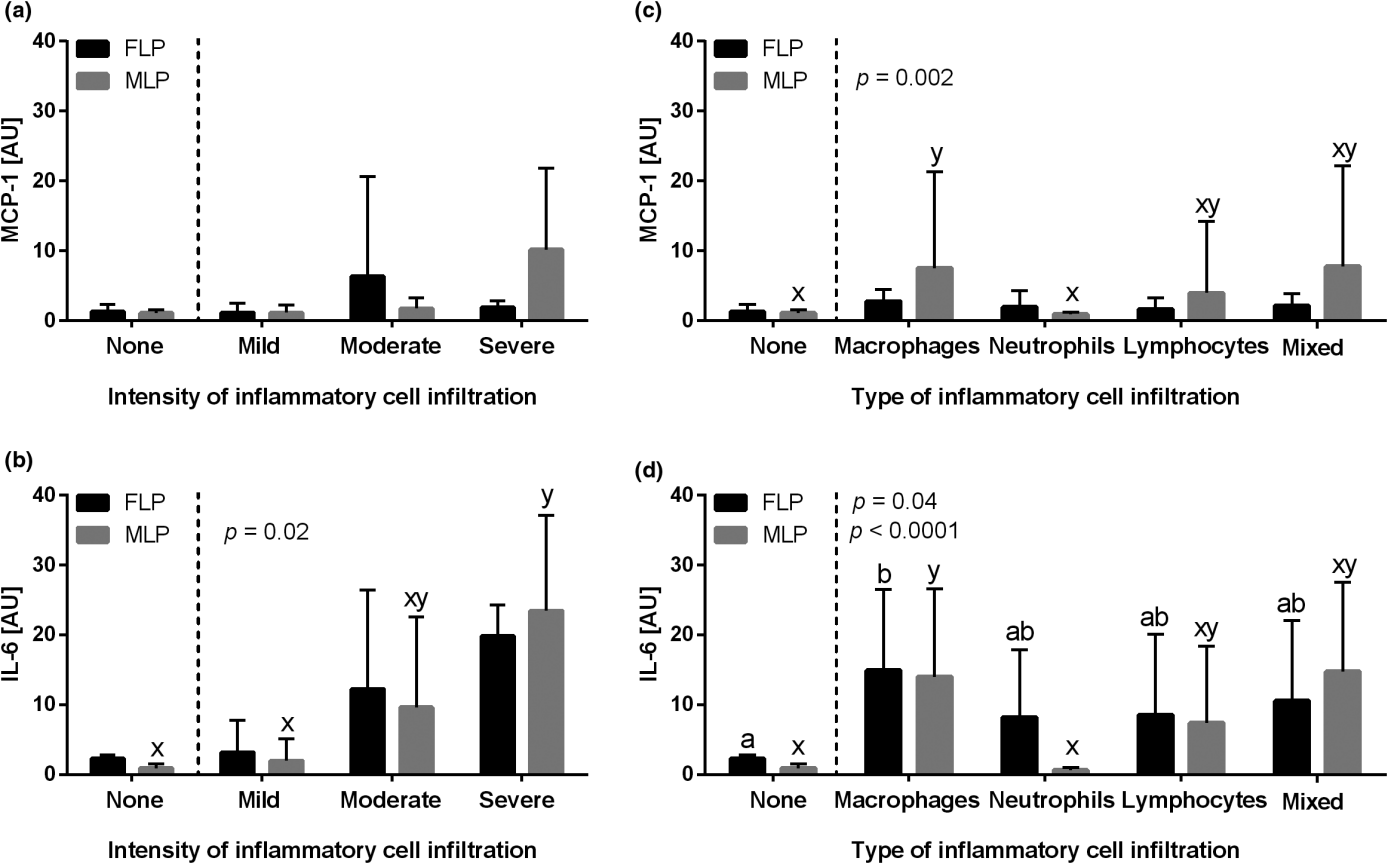
The transcription levels (mean + SD) of (a, c) the monocyte chemoattractant protein‐1 (*MCP‐1*) and (b, d) interleukin‐6 (*IL‐6*) in the mares' endometrium in follicular (FLP) or mid‐luteal (MLP) phases of the oestrous cycle. The endometrial samples evaluated with respect to the intensity of inflammatory cell infiltrate (none, mild, moderate and severe) are shown in graphs a and b, and according to the type of inflammatory cell infiltration (macrophages, neutrophils, lymphocytes and mixed cells) or no cell infiltration detected (none) in c and d. Different letters indicate differences between groups for *p *< .05

When the phases of the oestrous cycle were considered, the transcription of *MCP‐1* gene did not differ in equine endometrium depending on the intensity of inflammatory cell infiltration (FLP *p *= .253, MLP *p *= .142, Figure 5a), whereas the transcription of *IL‐6* gene increased gradually with the intensity infiltration in MLP (*p *= .02) but not in FLP (*p *= .1). In MLP compared to the control group, *IL‐6* gene expression was higher in the severe‐intensity infiltration group. No differences were noted among control, mild and moderate groups as well as between moderate and severe groups (Figure 5b). Furthermore, the transcription of both *MCP‐1* (MLP *p *= .002, Figure 5c) and *IL‐6* (FLP *p *= .04, MLP *p *< .0001, Figure 5d) genes was higher in equine endometrium with macrophages infiltration than in the control groups in both FLP and MLP, excepting *MCP‐1* in FLP (*p* = .2). For the transcription of *MCP‐1* in MLP and *IL‐6* in FLP, no differences were noted among control group, the neutrophil, lymphocyte and mixed types of infiltration, as well as among the macrophage, lymphocyte and mixed types of infiltration. However, for the transcription of *IL‐6* in FLP, no differences were noted among control group, the neutrophil, lymphocyte and mixed types of infiltration, and also among macrophage, neutrophil, lymphocyte and mixed types of infiltration. No differences were found between the phases of the oestrous cycle for any of the studied proinflammatory molecules (*p *> .05).

**FIGURE 6:**
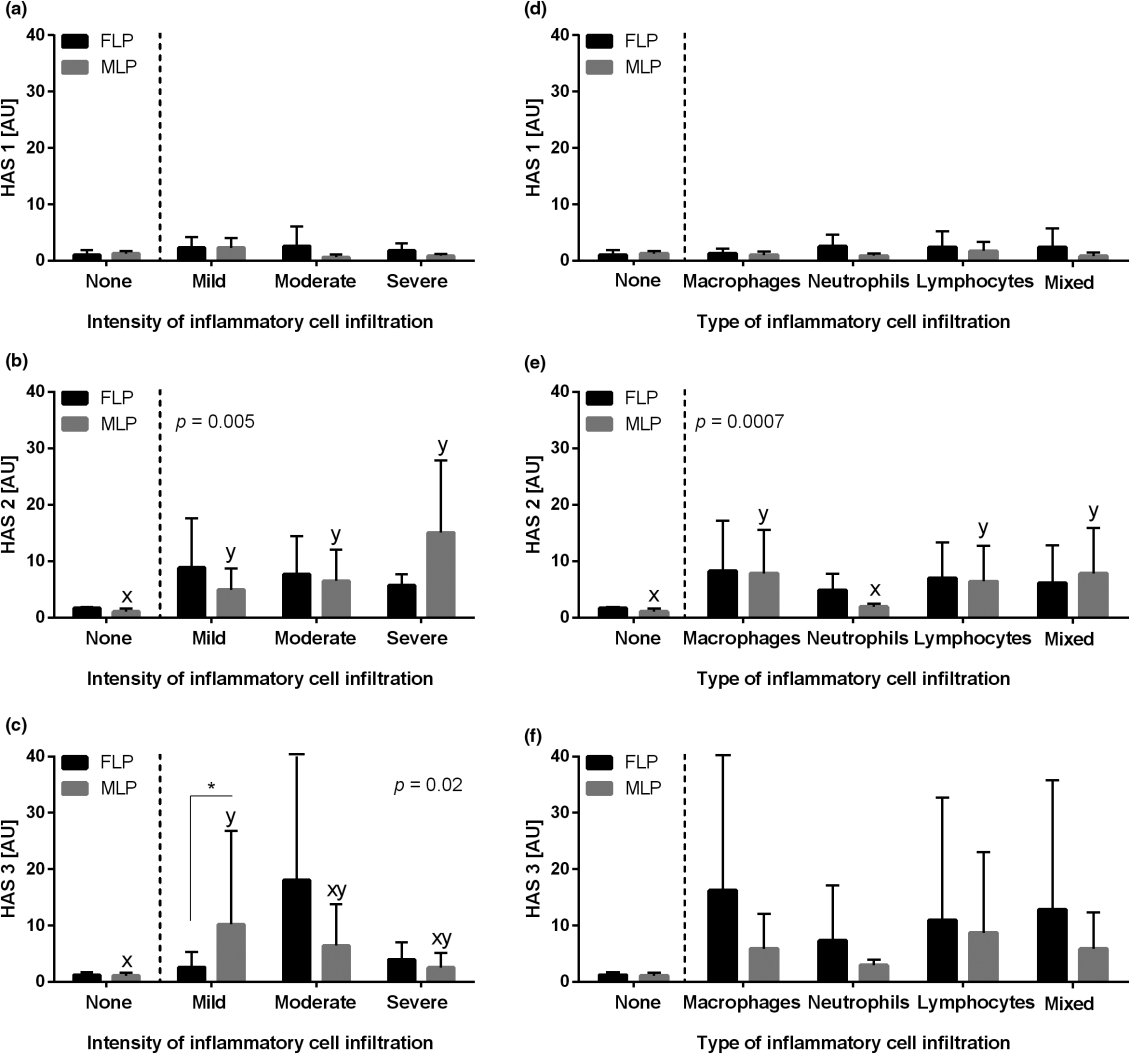
The transcription levels (mean + SD) of (a, d) the hyaluronan synthase 1 (*HAS 1*), (b, e) hyaluronan synthase 2 (*HAS 2*) and (c, f) hyaluronan synthase 3 (*HAS 3*) in the mares' endometrium in follicular (FLP) or mid‐luteal (MLP) phases of the oestrous cycle evaluated with respect to the intensity of inflammatory cell infiltrate (none, mild, moderate and severe) are shown in graphs a, b and c, and according to the type of inflammatory cell infiltration (macrophages, neutrophils, lymphocytes and mixed cells) or no cell infiltration detected (none) in d, e and f. Different letters indicate differences between groups for the same oestrous cycle phase (*p *< .05). The asterisk indicates differences between phases of oestrous cycle (*p < .05)

When the phases of the oestrous cycle were considered, the transcription of *HAS 1* gene did not differ in equine endometrium depending on the intensity of inflammatory cell infiltration (FLP *p *= .6, MLP *p *= .05, Figure 6a) and the type of inflammatory cell infiltration (FLP *p *= .5, MLP *p* = .2, Figure 6d). In addition, the transcription of *HAS 2* gene was higher in equine‐infiltrated endometrium than in the control group regardless of the intensity infiltration in MLP (*p *= .005) but not in FLP (*p *= .2). No differences among mild, moderate and severe groups were found (Figure 6b). Similarly, in MLP, the transcription of *HAS 3* gene was higher in equine endometrium with the mild‐intensity infiltration than in the control group, but not in the moderate and severe groups (*p *= .01). No other differences were noted for *HAS 3* gene expression among control, moderate and severe groups, as well as mild, moderate and severe groups (Figure 6c). The transcription of *HAS 3* gene in FLP did not differ depending on the intensity of inflammatory cell infiltration (FLP *p *= .3, Figure 6c). The transcription of *HAS 2* genes in MLP was higher in equine endometrium with macrophages, lymphocytes and mixed inflammatory cell infiltration than in the control and neutrophils groups (*p *= .0007, Figure 6e). However, the transcription of *HAS 2* gene in FLP (*p* = .3, Figure 6e), *HAS 3* gene in FLP (*p *= .5, Figure 6f) and *HAS 3* gene in MLP (*p *= .06, Figure 6f) did not differ depending on the type of inflammatory cell infiltration. Additionally, the transcription of *HAS 3* gene in the mild‐intensity infiltration group was higher in MLP than in FLP (*p *= .04). No other differences (*p *> .05) were found between the phases of the oestrous cycle for the studied hyaluronan synthases.

## 4 DISCUSSION

As shown in recent studies, NF‐κB‐dependent pathway may take part in the development of endometrosis. Its activation leads to inhibition of IL‐6 and activation of hyaluronan synthases, HAS 1 and HAS 3. These changes are especially prominent in the follicular phase of the oestrous cycle, and the destructive type of endometrosis, characterized by extensive glands destruction and degeneration. Additionally, levels of transcripts of genes of immunomodulatory cytokines depend on the stage of the oestrous cycle, with an increase in *MCP‐1* and a decrease in *IL‐6* in the follicular phase, which may result in increased cell migration into the endometrium (Domino et al., 2020). Importantly, equine endometrial samples used in the previous studies (Domino et al., 2020) did not show signs of inflammation. While these cytokines are thought to be involved in the endometrosis pathway, they are essentially important in inflammation development (Christoffersen & Troedsson, 2017; Woodward et al., 2013; Marth et al., 2018; Skarzynski et al., 2020; Morris et al., 2020; Hedia et al., 2021). This indicates the importance of NF‐κB evaluation in the context of endometritis. Research conducted regarding the type and severity of inflammation should give an insight into the role of this pathway in the regulation of modulatory cytokines. Differences in the regulation of this pathway may suggest separate mechanisms in the pathogenesis of endometrosis and endometritis and give another perspective on the influence of inflammation on the development of endometrosis, which has been widely studied (Aresu et al., 2012; Rebordão, Carneiro, et al., 2014; Rebordão, Galvão, et al., 2014; Klose & Schoon, 2016; Rebordão et al., 2018; Schöniger & Schoon, 2020; Skarzynski et al., 2020)

An increase in the transcription of the *MCP‐1* gene in moderate and severe infiltration may suggest that its function is rather sustained in the inflammatory process, than its initiation. The highest transcription was found in macrophages‐dominant infiltration. While MCP‐1 is being produced by this type of cell, it also increases its further migration into the tissue, which might explain these results. This can also confirm the major regulatory function of macrophages, which are responsible for the production of a variety of cytokines (Arici et al., 1999; Fumuso et al., 2003; Yadav et al., 2010). Interestingly, MCP‐1 was found not being connected with the fibrosis process in the endometrium during endometrosis (Domino et al., 2020), except in the inactive destructive endometrosis in the follicular phase (Jasiński et al., 2021). While this cytokine production may be regulated by the NF‐κB pathway, in the current study this was not in agreement with the transcript levels of NF‐κB subunits. This result may suggest a different role of this cytokine in the regulation in endometritis and endometrosis establishment, especially regarding MCP‐1 involvement (Yadav et al., 2010; Domino et al., 2020; Jasiński et al., 2021).

Transcription of *IL‐6* gene increased along with the increase in inflammation severity, which is in line with recent studies, as it is one of the most important cytokines in endometritis development (Fumuso et al., 2003; Woodward et al., 2013; Christoffersen & Troedsson, 2017; Skarzynski et al., 2020). An increase in transcription was found in macrophage‐ and mixed‐type inflammatory cell infiltration. These results confirm that macrophages are the main cells responsible for the production of this cytokine. Thus, its regulatory function for cell migration and infiltration is crucial in the inflammatory response (Arici et al., 1999; Yadav et al., 2010). In PBIE, cytokine production was found to be prolonged in susceptible mares, making them less fertile, as endometritis persists at the time of embryo descent from the oviduct to the uterus. Current results may confirm that macrophage‐related regulation is an important factor in prolonged inflammation, and is a reason for decreased fertility (Christoffersen et al., 2012; Tanaka et al., 2014; Christoffersen & Troedsson, 2017; Morris et al., 2020; Skarzynski et al., 2020). Transcription levels of *IL‐6* and *MCP‐1* genes in inflammation differ from those in endometrosis, where none of them was involved in the severity of endometrial fibrosis degenerative processes. This may suggest that their function in inflammation is not affecting fibrotic processes in ECM (Domino et al., 2020; Jasiński et al., 2021).

Lack of significant differences in transcription of *HAS 1* gene suggests that this enzyme may not be involved in cell migration and adhesion in mare endometrial inflamed tissue. Nevertheless, the transcription of *HAS 2* and *HAS 3* genes has raised with increased immune cell infiltration in the endometrium. Changes were found in all types of the infiltrate. Yet, for both genes, the increase in transcript levels was the lowest in neutrophil infiltration. This result is consistent with the fact that binding of CD44, which is the receptor for hyaluronic acid, with its ligand occurs during migration of T‐lymphocytes and monocytes. Fragmented hyaluronic acid acts as a proinflammatory particle, increasing cytokine production (Kessler et al., 2008). This is compatible with the achieved results, suggesting hyaluronan role in leukocyte migration into the equine endometrium. In samples with endometrosis, transcription of *HAS 1* gene was increased in the initial stage of the disease, while transcription of *HAS 3* gene was higher in all groups of endometrosis compared to unaffected endometrium. On the contrary, transcription of *HAS 2* gene did not differ between endometrosis and healthy endometrium, but its transcription increased in active destructive endometrosis form during oestrus, in which severity and intensity of changes are the highest (Jasiński et al., 2021). This shows that transcription of different synthases might be engaged in endometrosis and endometritis.

The present data show that the NF‐κB pathway is not taking part in the regulation of cell migration in the equine endometrium. The increase in transcription of genes of selected cytokines and enzymes is not in line with the results considering NF‐κB subunits. This is an important finding, suggesting that their synthesis, in the equine endometrium, is not regulated by this pathway, but different mechanisms are involved. This finding may be important in studies regarding equine endometrosis. In a previous study, we have found that hormone‐dependent activation of NF‐κB may lead to fibrosis (Domino et al., 2020). Therefore, one may suggest that there is a specific pathway regulating only fibrosis in mare’s uterus (endometrosis), but not endometritis. This suggests that it may be a specific marker for degeneration and fibrosis in endometrosis, possibly useful in diagnosis and progression of the severity of the process. It is especially important in uterine clearance after mating or artificial insemination. Uterine immune cells infiltration makes it possible to prepare the intrauterine environment for embryo descent and develop pregnancy (Fumuso et al., 2003; Christoffersen et al., 2012; Skarzynski et al., 2020). Proper functioning of the uterine immune system is not only important in case of injury or infection but it is also crucial for the maintenance of mare’s fertility, by enabling physiological functioning of the uterus, regulating endometrial hormone production, especially by cytokines (Skarzynski et al., 2020).

In a regularly cycling mare, the number of immune cells in the endometrium varies depending on the phase of the oestrous cycle (Da Costa et al., 2003; Fumuso et al., 2003; Gerber, 2006), to which the current results are in agreement as the majority of the transcription differences were showed only in the mares’ endometrium in the mid‐luteal phases compared with the mares’ endometrium in the follicular phase. Yet, in increased leukocyte infiltration in the endometrium, migration of cells, as well as their defensive functions, such as phagocytosis, are not depending on the oestrous cycle phase (Da Costa et al., 2003; Fumuso et al., 2003; Gerber, 2006). This fact may explain poor correlations when the phase of the oestrous cycle was taken into consideration. Immune system competencies are therefore not disturbed by physiological changes in ovarian steroids levels (Gerber, 2006). However, other NF‐κB‐signalling pathway seems to be activated in the follicular phase and in the mid‐luteal phase in the presence of inactive destructive or active destructive endometrosis respectively (Jasiński et al., 2021). On the other hand, concerning the classical Kenney and Doig classification of endometrosis (Kenney & Doig, 1986), greater differences were found in the follicular phase than in the luteal phase of the oestrous cycle (Domino et al., 2020). In the current study, no oestrous cycle‐related differences were found in the NF‐κB subunits of both canonical and non‐canonical pathways, as well as more differences in transcription of *HAS2*, *HAS3*, *MCP‐1* and *IL‐6* were noted when compared non‐divided than oestrous cycle‐divided data series. Therefore, one may assume that inflammatory processes have taken over the cell cytokine profile. However, further studies are still required to prove reported assumptions.

## 5 CONCLUSIONS

In mare endometrium, transcription of *IL‐6* and *MCP‐1* genes increased with the severity of inflammation, especially with dominant macrophages. Among hyaluronan synthases, transcription of *HAS 2* and *HAS 3* genes was higher in the presence of endometritis, possibly increasing cell migration and adhesion. Yet, their production was not regulated by the NF‐κB pathway, suggesting this via is not engaged in endometritis regulation. This claim shows the difference with data obtained on endometrosis, suggesting a separate pathway for the development of the disease. In addition, oestrous cycle did not affect the expression of the genes under study, suggesting that inflammation takes over cytokine control of immune cells.

## CONFLICT OF INTEREST

None of the authors have any conflict of interest to declare.

## AUTHORS CONTRIBUTION

The idea for the study was conceived by TJ and MD. The experimental protocol was designed by TJ, LZ, EK and MD. The data were obtained by TJ, LZ, EK, EJK, GFD and MD. It was statistically analysed by TJ, GFD and MD and discussed by all authors. The manuscript was written by TJ, LZ and MD, critically revised by GFD and finally revised and accepted by all authors.

## ETHICAL APPROVAL

The Ethics Review Board II Local Committee for Ethics in Animal Research of Warsaw University of Life Sciences – SGGW considers that this type of project does not fall under the legislation for the protection of animals used for scientific purposes, national decree‐law (Dz. U. 2015 poz. 266 and 2010‐63‐EU directive).

## Data Availability

The data that support the findings of this study are available from the corresponding author upon reasonable request.
